# A case report of multiple endocrine neoplasia type 1 and autoimmune disease: Coincidence or correlation?

**DOI:** 10.1097/MD.0000000000028145

**Published:** 2021-12-10

**Authors:** Carolina Chaves, Tiago Nunes da Silva, Bernardo Dias Pereira, João Anselmo, Isabel Claro, Branca M. Cavaco, Ana Saramago, Valeriano Leite

**Affiliations:** a Serviço de Endocrinologia e Nutrição, Hospital Divino Espírito Santo de Ponta Delgada, Azores Islands, Portugal; b Serviço de Endocrinologia, Instituto Português de Oncologia de Lisboa Francisco Gentil, Lisbon, Portugal; c Unidade de Investigação em Patobiologia Molecular (UIPM), Instituto Português de Oncologia de Lisboa Francisco Gentil, Lisbon, Portugal; d Serviço de Gastroenterologia, Instituto Português de Oncologia de Lisboa Francisco Gentil, Lisbon, Portugal; e Nova Medical School, Lisbon, Portugal.

**Keywords:** autoimmunity, genetics, multiple endocrine neoplasia, mutations

## Abstract

**Rationale:**

Multiple Endocrine Neoplasia type 1 (MEN1) is a familial syndrome that results from the disruption of a tumor suppressor protein called MENIN. Its management is challenging, as MEN1 affects different endocrine tissues and predisposes to both benign and malignant tumors. MENIN-deficient cells have recently been recognized to play a role in triggering autoimmunity. Herein, we present a case of MEN1 with multiple endocrine and autoimmune disorders.

**Patient concerns:**

A 50 years old female with a 25 years history of complicated nephrolithiasis presented with primary hyperparathyroidism.

**Diagnoses:**

Over several decades, she was diagnosed with recurrent primary hyperparathyroidism, autoimmune thyroiditis, multinodular goiter, pernicious anemia, metastatic gastric type 1 neuroendocrine tumor, macroprolactinemia, gonadotropin deficiency, mucosa-associated lymphoid tissue lymphoma of the thyroid gland, positive anti-calcium sensor receptor antibodies, and *BRCA 1/2*-negative invasive breast cancer. The autoimmune regulator gene was sequenced, but no pathogenic variants were found. Next-generation sequencing revealed both a pathogenic *MEN1* mutation and a benign *CDC73* gene variant. Familial genetic screening revealed a large kindred with multiple carriers of one or both genetic variants (*MEN1* = 19; *CDC73* = 7).

**Interventions:**

The patient underwent surgical excision of three parathyroid glands, total thyroidectomy and breast tumorectomy plus tamoxifen, and monthly injections of octreotide. The patient and family members with the *MEN1* mutation are under a life-long surveillance program for MEN1 prototypic tumors.

**Outcomes:**

The patient was stable and alive during a 24-years follow-up period.

**Lessons:**

With the present case, the authors highlight a new interplay between MENIN and the immune system, which may have implications for future targeted life-long surveillance and treatment of MEN1 patients.

## 1 Introduction

MENIN is a ubiquitous product of *MEN1*, which acts as a tumor suppressor protein.^[1]^ If mutated, it leads to the development of several neuroendocrine tumors, usually in the parathyroid gland, pituitary gland, and gastroenteropancreatic neuroendocrine tissues. Primary hyperparathyroidism is usually the first manifestation detected and has high penetrance in individuals with MEN1, even at younger ages.^[2]^ Pituitary tumors are present in 40% of patients with MEN1 and are usually prolactin-producing or non-functioning tumors.^[3]^ Type II gastric neuroendocrine tumors (NETs) are associated with MEN1, often affecting both the fundus and body of the stomach. Most often, they are asymptomatic and are found in the workup of anemia or symptoms of peptic ulcers.^[4]^ According to the Universal Mutation Database, 1698 different germline and somatic mutations of the *MEN1* gene are known,^[5]^ but it has not been possible to establish a genotype–phenotype correlation. Exogenous and endogenous factors, in addition to the *MEN1* mutation, may be involved in the tumorigenic mechanism behind MEN1, giving rise to distinct phenotypes.^[6]^ Although the loss of MENIN expression is a *sine qua non* condition for the development of MEN1-associated tumors, *in silico* functional analysis showed that variants in genes involved in the same proliferative and apoptotic tumorigenic pathways may act as modifiers, including Kirsten rat sarcoma virus (KRAS), Wnt Family Member 2B (WNT2B), Interleukin 3 Receptor Subunit Alpha (IL3RA) and TNF Receptor Superfamily Member 10A (TNFRSF10A). These genes have the potential to be *MEN1* modifiers, as MENIN has been shown to repress MAPK-driven proliferation downstream of KRAS, control WNT signaling through interaction with β-catenin, regulate expression of interleukins, and promote TNF-α-induced apoptosis through upregulation of caspase 8.^[7]^ In recent years, experimental data revealed a new possible role of MENIN in autoimmunity, in which MENIN-deficient CD4 and CD8 lymphocytes are prone to dysfunction and trigger autoimmune disorders.^[8,9]^ This new evidence may have important implications for MEN1 patients, including the screening for and treatment of autoimmune diseases in their lifelong surveillance programs. Herein, we report an unusual patient with multiple endocrine, autoimmune, and oncological diseases that may be linked to the role of MENIN in regulating both the endocrine and immune systems. We also present the patient's pedigree in an attempt to bring light to such a rare clinical case.

## 2 Case presentation

We describe the case of a 79-years-old female patient born in the Azores Islands, living in mainland Portugal since her twenties, who was diagnosed with recurrent symptomatic hydronephrosis due to left nephrolithiasis in 1969, at the age of 25 years. She had no relevant past or family history. After 15 years of recurrent symptomatic hydronephrosis, the patient underwent unilateral nephrectomy. At the age of 50 years, she began to be followed at our institution. The diagnosis of multinodular goiter and primary hyperparathyroidism was established, with serum calcium (sCa) of 11.5 mg/dL (normal range: 8.4–10.2), parathyroid hormone (PTH) of 264 pg/mL (normal range: 10–60), and normal 25- and 1,25-hydroxyvitamin D. She was also diagnosed with autoimmune pernicious anemia with positive anti-intrinsic factor antibodies and anti-parietal cell antibodies, and primary autoimmune hypothyroidism with high titers of anti-thyroperoxidase antibodies (>10,000 IU/mL; normal range: <35). She was started on levothyroxine 50 μ/day, oral vitamin B12, and iron supplements. Fine-needle aspiration of the multinodular goiter revealed lymphocytic thyroiditis. Due to hypercalcemia and multiple thyroid nodules, she underwent subtotal thyroidectomy, leaving a remnant of the left lobe as well as right and left inferior parathyroidectomy. Histology confirmed the presence of Hashimoto's thyroiditis and parathyroid adenoma with lymphocytic infiltration. At 58 years of age, she underwent esophagogastroduodenoscopy due to dyspepsia, which showed multiple small (>20) red, sessile polyps in the gastric body and fundus. Biopsy of the lesions revealed a well-differentiated G1 NET (Ki-67 of 1%) in the body of the stomach, with associated lesions of chronic atrophic gastritis and intestinal metaplasia. Computed tomography (CT) revealed bilateral metastatic lung lesions, which showed high uptake in the [111In]-octreotide scintigraphy. CT-guided lung biopsy of these lesions confirmed a neuroendocrine origin. Increased serum levels of chromogranin A were documented (820 nmol/L; normal range: <45), and gastrin levels were also high (1138 pg/mL; normal range <90). The patient was started on octreotide (20 mg/month). At this point, MEN1 was suspected, and biochemical screening of pituitary function revealed a high serum prolactin level of 56 ng/mL (normal range < 20) and inappropriately low FSH (<0.1 UI/L) and LH (<0.3UI/L) levels in menopausal women. Further investigation revealed that hyperprolactinemia was due to macroprolactinemia (recovery of monomeric prolactin after precipitation with polyethylene glycol: 9%; normal >40%).^[10]^ The remaining pituitary function and pituitary magnetic resonance imaging (MRI) scan were normal. A presumptive diagnosis of autoimmune hypophysitis has been proposed. After informed consent was obtained from the patient, genetic *MEN1* screening performed with single-strand conformational polymorphism (SSCP) was negative. At 61 years of age, hypercalcemia recurred, and a lesion in the topography of the left upper parathyroid was found by technetium-99m sestamibi scintigraphy. Three years later, due to an increase in sCa (12.2 mg/dL) and PTH (191 pg/mL), the patient underwent removal of the left upper parathyroid, along with an enlarged left thyroid lobe. Histological examination showed an 18-mm parathyroid adenoma with lymphocytic infiltration and low-grade mucosa-associated lymphoid tissue (MALT) B lymphoma of the thyroid gland. Serum anti-calcium sensor receptor (CaSR) antibodies were positive. A few months later, the patient underwent urgent cholecystectomy for acute cholecystitis, probably because of the prolonged use of somatostatin analogs. She remained under surveillance without any new manifestations until she was 68 years old when, due to abnormal uterine bleeding, she underwent a pelvic ultrasound that revealed two large uterine leiomyomas. At 69-years-old the patient was diagnosed with a left breast invasive carcinoma and underwent successful surgery followed by tamoxifen therapy, which was later discontinued. At this time, due to the high clinical probability of MEN1, after further informed consent from the patient her leukocyte DNA was analyzed by next-generation sequencing (TruSight Cancer Sequencing panel, Illumina), and a novel heterozygous germline mutation in exon 9 of the *MEN1* gene (c.1321_1323dup) was found, which is expected to lead to a duplication of the tryptophan residue (p.Trp441dup) of MENIN. This variant was absent from controls in the databases ALFA Allele Frequency, 1000 Genomes Project, or ExAC. *In silico* analysis performed using the Mutation Taster software tool classified this variant as disease-causing. The altered amino acid is localized to a highly conserved site during evolution. Based on the guidelines of the American College of Medical Genetics and Genomics (ACMG), this variant is classified as likely pathogenic.^[11]^ NGS analysis also detected a variant in the Cell Division Cycle 73 (*CDC73*) gene (c.-4_-11insG). This variant is described in the database ALFA Allele Frequency and gnomAD exomes, with an allele frequency of 0.21% (European population) and 1.02% (non-Finnish European), respectively. The *in silico* analysis performed using the Mutation Taster software tool classified this variant as disease-causing. Sequence alignment of *CDC73* encoding the proximal 5’UTR revealed that this region showed limited phylogenetic conservation. This variant has eight submissions in ClinVar: benign (5) and uncertain significance (3). Based on the ACMG guidelines, this variant is classified as benign.^[11]^ Due to the multiple autoimmune conditions of this patient (pernicious anemia, lymphocytic thyroiditis, macroprolactinemia, possible lymphocytic hypophysitis, anti-CaSr antibodies), the autoimmune regulator (*AIRE*) gene was sequenced, but no pathogenic mutations were found. Breast cancer (*BRCA*) genes *1/2* testing was negative. Recently, a ^68^Gallium-DOTANOC position emission tomography/CT showed two foci of increased uptake in the pancreatic body and tail. The patient refused abdominal MRI due to claustrophobia, and an abdominal CT scan did not detect any pancreatic lesions. Since then, she has been clinically stable, without evidence of recurrent primary hyperparathyroidism or progression of her metastatic gastric neuroendocrine tumor. The patient was kept under octreotide 20 mg/month because of intolerance to higher doses.

**Figure 1:**
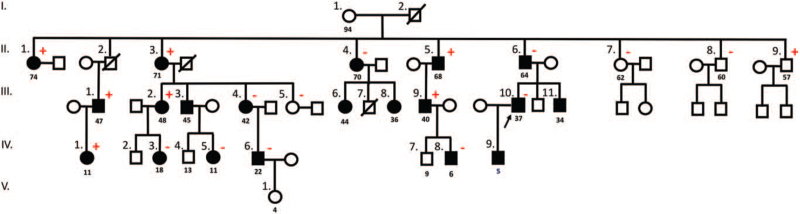
Family tree of the MEN1 kindred. CaSR, calcium sensor receptor; *CDC73,* Cell Division Cycle 73 gene; MALT, mucosa-associated lymphoid tissue; *MEN1,* Multiple Endocrine Neoplasia type 1 gene; NET, neuroendocrine tumor. Symbols: • 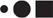, *MEN1* carriers; plus (in red), *CDC73* variant positive carriers; minus (in red), *CDC73* variant negative carriers; II.1., member with recurrent primary hyperparathyroidism, metastatic type 1 gastric NET, pernicious anemia, macroprolactinemia, autoimmune thyroiditis, thyroid MALT B lymphoma, and positive anti-CaSR antibodies; II.2., member with metastatic renal cell carcinoma (deceased in 2006, at 61 years old) and idiopathic hypercalcemia (calcium 10.9–11.6 mg/dL); II.3., member with primary hyperparathyroidism; II.4., member with primary hyperparathyroidism, adrenal adenoma, autoimmune hypothyroidism and small intestine adenocarcinoma; II.5., member with multiple (2) non-functioning pancreatic NETs and non-functioning typical pulmonary carcinoid; II.6., member with non-functioning pancreatic NET and laryngeal carcinoma; III.1., member with multiple (3) non-functioning pancreatic NETs; III.2., member with recurrent primary hyperparathyroidism and non-functioning pancreatic NET; III.3., member with primary hyperparathyroidism, non-functioning typical pulmonary carcinoid and non-functioning pancreatic NET; III.4., member with primary hyperparathyroidism; III.6., member with primary hyperparathyroidism, idiopathic hyperprolactinemia and epilepsy; III.7., member who died at 19 years old (car accident); III.8., member with primary hyperparathyroidism and sinusitis; III.9., member with primary hyperparathyroidism; III.10. member with primary hyperparathyroidism and microprolactinoma; III.11., member with multiple (2) non-functioning pancreatic NET; IV.1., asymptomatic member; IV.3., member with Cushing's disease and uterine myoma; IV.5., asymptomatic member; IV.6., asymptomatic member; IV.8., asymptomatic member; IV.9., member not yet screened for MEN1 prototypic tumors. Note: The age of genetic diagnosis of MEN1 is presented below the symbol of each affected family member.

Later, we found that the patient's family, living in the Azores islands and with whom the patient had no social contacts, was under a surveillance program for MEN1 prototypic tumors in another institution, where 19 members were carriers of the same mutation in our patient (Fig. 1). The institution where these family is being followed provided the informed consent for genetic screening of each patient, which also includes consent for anonymous scientific report of his/her own clinical data. The *CDC73* variant found in our patient was also present in seven family members, including carriers and non-carriers of the *MEN1* mutation (Fig. 1). To date, the most frequent manifestation in this pedigree was primary hyperparathyroidism (n/%: 10/50%) diagnosed with a median age of 44 years (minimum-maximum: 36–70). The youngest patient was diagnosed at 36 years of age and was the only member who underwent subtotal parathyroidectomy with autologous implantation of parathyroid tissue in the left forearm due to complicated nephrolithiasis. Histology revealed hyperplasia of the parathyroid glands with lymphocytic infiltration of the surrounding thyroid tissue (he presented with normal thyroid function, but thyroid autoantibodies were both positive). We studied the prevalence and phenotype of family members who manifested both genetic alterations (*MEN1* and *CDC73*); individuals with both alterations presented with a prevalence of hyperparathyroidism of 50% (3/6) compared to 55% (6/11) with *MEN1* mutation alone. The second most frequent manifestation in this family was non-functioning pancreatic NET (n/%: 6/31.5%) diagnosed with a median age of 49 years (minimum-maximum: 34–64), and 3 cases (50%) of multiple tumors. Pituitary lesions were the third most common manifestation, presenting in 2 patients (10%)—microprolactinoma and Cushing's disease—and diagnosed with a median age of 27 years old (minimum-maximum 16–37). Other manifestations found to date were non-functioning typical bronchial carcinoid (2/10%), adrenal adenoma (1/5%), uterine myoma (1/5%), idiopathic hyperprolactinemia (1/5%), and small intestine adenocarcinoma (1/5%). Two additional family members had autoimmune thyroiditis (Fig. 1).

## 3 Discussion

This is a rare and interesting clinical report of an individual with MEN1 and a constellation of neuroendocrine, oncological, and autoimmune diseases: pernicious anemia with type 1 gastric NET and lung metastases, lymphocytic thyroiditis with concurrent MALT lymphoma, macroprolactinemia, and gonadotropin deficiency (possibly due to lymphocytic hypophysitis), parathyroid adenomas with lymphocytic infiltration and positive antiCaSR antibodies, and breast carcinoma. These multiple findings of targeted autoimmunity to endocrine tissues could not be explained by mutations in the *AIRE* gene, the cause of autoimmune polyglandular syndrome type 1, characterized by chronic candidiasis and several autoimmune diseases involving mainly endocrine tissues. Recurrent hyperparathyroidism and a gastric NET at a young age led to a putative clinical diagnosis of MEN1, but the genetic study performed in 2004 (SSCP) was negative for this condition.

The first clinical manifestation of the patient was hyperparathyroidism, which led to her first parathyroidectomy in her fifth decade of life. As MEN1 was initially ruled out, another genetic cause of primary hyperparathyroidism and hypercalcemia was identified. Using NGS, both an *MEN1* mutation and a *CDC73* gene variant were identified. Family genetic screening showed that some members of her family (Fig. 1) also had both genetic alterations. To the best of our knowledge, there are no data in the literature regarding synchronous mutations of *MEN1* and *CDC73* occurring in the same patient/kindred. As stated in the previous section, we found no differences in phenotypic features between carriers of *MEN1* and *MEN1/CDC73* mutations. The surveillance program for prototypic tumors of MEN1 in our patient's family did not change in terms of including imaging to prototypic tumors of carriers of *CDC73* mutations other than parathyroid disease, as the *CDC73* variant detected in some family members is probably benign. It is noteworthy that in one family with hyperparathyroidism-jaw tumor syndrome (HPT-JT),^[12]^ the c.-4_-11insG 5’UTR variant was found to be in *cis* with a deletion of exons 4 to 10 of *CDC73*. In vitro assays of 5’UTR insertion have been shown to impair the function of *CDC73* mRNA. Western blot analysis of cell protein extracts showed reduced levels of exogenous parafibromin in cells transfected with the MUT-5’UTR-Flag construct as compared with the WT-5’UTR-Flag construct. However, based on limited phylogenetic conservation and conflicting interpretations of variant pathogenicity described in ClinVar (benign, likely benign, uncertain significance),^[13]^ it was suggested that the affected status was due to the large deletion, without any attributable influence of the 5’UTR variant. Our results support the benign nature of this *CDC73* variant, as individuals with both alterations presented a prevalence of hyperparathyroidism of 50% (3/6), similar to the 55% (6/11) of family members with only the *MEN1* mutation. Furthermore, the only family member (II. 9.; Fig. 1) without *MEN1* mutations harboring the *CDC73* variant is asymptomatic.

At 59 years of age, esophagogastroduodenoscopy with biopsy revealed a well-differentiated G1 NET in the stomach, with associated lesions of chronic atrophic gastritis. As the patient had NET-derived lung metastasis and concomitant functional imaging did not detect suspicious lesions in other organs, we assumed the gastric NET to be the primary tumor. To the best of our knowledge, this is the only case of type 1 gastric NET with pulmonary metastasis reported in the literature. A literature review carried out so far reveals that G1 NET tumors either do not metastasize at all, or have a low rate of nodal metastases, and the risk of distant metastases seems to be higher in patients with polyps larger than 20 mm, which was not the case in the patient described herein.^[14]^ Thirteen years after right and left inferior parathyroidectomy, the patient underwent a third parathyroidectomy, and a parathyroid adenoma with lymphocytic infiltration associated with low-grade MALT B lymphoma was diagnosed. Interestingly, lymphocytic infiltration of the parathyroid glands is a very rare finding,^[15]^ in addition to positive anti-CaSR antibodies. Additionally, a low-grade MALT B lymphoma of the thyroid gland was diagnosed during left lobectomy. Primary thyroid lymphomas have an estimated annual incidence of 2 cases per million inhabitants, and women are more commonly affected than men (2–8:1), with the typical age at presentation being between the sixth and seventh decades of life. Most thyroid lymphomas are non-Hodgkin lymphomas of B-cell origin, and MALT lymphomas represent 10% to 23% of these cases. The risk of developing thyroid lymphoma is increased in patients with Hashimoto's thyroiditis, as in our patient, with a relative risk of 67% compared to those without thyroiditis.^[16]^ MALT B lymphoma has been previously described in a MEN1 patient with colon MALT lymphoma after several manifestations of this syndrome.^[17]^ Despite its well-known role as an endocrine tumor suppressor, MENIN was recently implicated as an oncogenic promoter in the hematopoietic system, where it acts as a fundamental link between mixed-lineage leukemia histone methyltransferase and lens epithelium-derived growth factor, a chromatin-associated protein coactivator that was previously implicated in promoting leukemia and autoimmunity.^[18]^ Finally, the hyperprolactinemia diagnosed in our patient was due to macroprolactinemia, a complex of monomeric prolactin bound to immunoglobulin G, which has been described in patients with other autoimmune diseases.^[10]^ The patient also had gonadotropin deficiency but normal pituitary MRI, ruling out the presence of a pituitary adenoma and suggesting pituitary hypophysitis as a likely cause of hypogonadism. With aging, CD4 and CD8 T lymphocytes may acquire a typical senescence secretory phenotype, which is characterized by a marked increase in the secretion of pro-inflammatory cytokines, extracellular matrix remodeling factors, and neoangiogenic factors. Through these aging secretory patterns, T cells may induce an increased susceptibility to autoimmune diseases. MENIN was found to prevent the evolution of this senescence-secretory phenotype of CD4 and CD8 T lymphocytes through modeling of mammalian target of rapamycin complex 1 activity and subsequent cellular metabolism. Thus, MENIN-deficient T lymphocytes evolve to a dysfunctional pattern prone to autoimmunity,^[8,9]^ and this may be the basis for the constellation of autoimmune manifestations present in our index patient.

Our patient also presented with breast cancer. A recent case–control study found a surprisingly high prevalence of breast cancer in MEN1 patients. In this Dutch survey, MEN1 patients had a median age at diagnosis of breast cancer of 45 years old, compared with 57.5 years old in female relatives without MEN1, and 61.2 years old in the Dutch sample population, suggesting the need for early screening of this condition in MEN1 female patients.^[19]^ Although we recognize it would be interesting to test this patient breast cancer tissue for somatic events in *the MEN1* gene, unfortunately, this is not possible because the patient had undergone surgery many years ago at another institution. Over three generations of her pedigree, no other cases of breast cancer were diagnosed, and no other family member presented such a large constellation of autoimmune manifestations.

In conclusion, the present case raises relevant issues regarding the putative role of MENIN in the autoimmune system. Future cumulative clinical data may shed light on the possible role of *MEN1* mutations (and type of mutations) in predisposing carriers to autoimmunity as well as for a certain phenotype, which may have implications for targeted life-long surveillance and treatment.

## Acknowledgments

We thank Dr Nicole Fabien, Director of the Immunology Laboratory of the Center Hospitalier Lyon-Sud, Hospices Civils de Lyon, Pierre-Bénite, France, for kindly performing the anti-CaSR antibody assay.

## Author contributions

**Conceptualization:** Carolina Chaves, Tiago Nunes da Silva, Bernardo Dias Pereira, Branca M. Cavaco, Ana Saramago, Valeriano Leite.

**Data curation:** Carolina Chaves, Valeriano Leite.

**Formal analysis:** Carolina Chaves, Tiago Nunes da Silva, Bernardo Dias Pereira, João Anselmo, Branca M. Cavaco, Ana Saramago, Valeriano Leite.

**Investigation:** Carolina Chaves, Tiago Nunes da Silva, Bernardo Dias Pereira, João Anselmo, Isabel Claro, Branca M. Cavaco, Ana Saramago, Valeriano Leite.

**Methodology:** Carolina Chaves, Tiago Nunes da Silva, Branca M. Cavaco, Ana Saramago, Valeriano Leite.

**Project administration:** Carolina Chaves, Tiago Nunes da Silva, Branca M. Cavaco, Valeriano Leite.

**Resources:** Branca M. Cavaco, Ana Saramago, Valeriano Leite.

**Supervision:** Tiago Nunes da Silva, João Anselmo, Branca M. Cavaco, Valeriano Leite.

**Validation:** Carolina Chaves, Tiago Nunes da Silva, Bernardo Dias Pereira, João Anselmo, Isabel Claro, Branca M. Cavaco, Ana Saramago, Valeriano Leite.

**Visualization:** Carolina Chaves, Tiago Nunes da Silva, Bernardo Dias Pereira, João Anselmo, Isabel Claro, Branca M. Cavaco, Valeriano Leite.

**Writing – original draft:** Carolina Chaves, Tiago Nunes da Silva, Ana Saramago.

**Writing – review & editing:** Carolina Chaves, Tiago Nunes da Silva, Bernardo Dias Pereira, João Anselmo, Isabel Claro, Branca M. Cavaco, Ana Saramago, Valeriano Leite.
